# The Bad Effects of Train-Catching

**Published:** 1881-05

**Authors:** 


### THE BAD EFFECTS OF TRAIN-CATCHING.

As the warm season approaches many of the denizens of the crowded cities take up their abode in the suburbs. The change necessitates daily trips on the railroad cars, a circumstance which, if properly managed, becomes a source of benefit. It is to be seriously regretted, however, that many unpleasant results follow the disposition on the part of too many communities to put off eating their breakfast as long as possible, running to catch the train, and will tarry at their counting house as long as possible, repeating the violent exercise in the evening. For the benefit of these "train catchers" it may be stated that authoritative cases were brought to the surface last year, through British channels, in which organic heart disease was traceable to the violent exercise incident to train-catching. Besides this, the most serious evils of indigestion are frequently engendered in this way. The rule that violent exercise should never be indulged in immediately after meals ought not be lost sight of by the class of persons for whose benefit this paragraph is intended.—*The Sanitary News.*

